# Short-Chain and Total Fatty Acid Profile of Faeces or Plasma as Predictors of Food-Responsive Enteropathy in Dogs: A Preliminary Study

**DOI:** 10.3390/ani12010089

**Published:** 2021-12-31

**Authors:** Cristina Higueras, Ana I. Rey, Rosa Escudero, David Díaz-Regañón, Fernando Rodríguez-Franco, Mercedes García-Sancho, Beatriz Agulla, Angel Sainz

**Affiliations:** 1 Department Animal Production, Animal Nutrition, Faculty of Veterinary Medicine, University Complutense of Madrid, Avda. Puerta de Hierro s/n, 28040 Madrid, Spain; crhiguer@ucm.es (C.H.); rmescude@ucm.es (R.E.); 2 Department Medicine and Surgery, Faculty of Veterinary Medicine, University Complutense of Madrid, Avda. Puerta de Hierro s/n, 28040 Madrid, Spain; drdiazreganon@ucm.es (D.D.-R.); ferdiges@ucm.es (F.R.-F.); mercgarc@ucm.es (M.G.-S.); angelehr@ucm.es (A.S.); 3 Department Medicina i Cirurgia Animals, Facultat de Veterinària, Universitat Autònoma de Barcelona, Bellaterra, 08193 Cerdanyola del Vàlles, Spain; beatriz.agulla.ba@gmail.com

**Keywords:** short-chain fatty acids, odd-chain fatty acids, long-chain fatty acids, dog, food-responsible enteropathy, gut health

## Abstract

**Simple Summary:**

Food-responsive enteropathy is the most common diagnosis given for dogs with chronic enteropathy, and there are no tests that can replace treatment trials. Furthermore, there is a lack of information on the specific nutritional status of these patients regarding the lipid profile that could relate them to the state of health/disease. This study evaluated differences in short-chain fatty acids and the total fatty acid profile of faeces and plasma as possible indicators of food-responsive enteropathy (FRE), as well as its relationship with body condition and the chronic enteropathy activity index. Changes in the long-chain fatty acid of plasma, and short-chain, branched and odd-chain fatty acids of faeces were detected in sick dogs, and high correlations were observed between some of these compounds and the existing calculated indices.

**Abstract:**

The aim of this study was to evaluate differences in short-chain fatty acids (SCFAs) and the total fatty acid profile of faeces or plasma as possible indicators of FRE in comparison with healthy dogs. FRE dogs had a lower concentration (*p* = 0.026) of plasma α-tocopherol as an indicator of the oxidative status of the animal, and lower C20:5n-3 (*p* = 0.033), C22:5n-3 (*p* = 0.005), polyunsaturated fatty acids (PUFA) (*p* = 0.021) and n-6 (*p* = 0.041) when compared with the control dogs; furthermore, sick dogs had higher proportions of plasma C20:3n-6 (*p* = 0.0056). The dogs with FRE showed a decrease in the production of faecal levels of SCFAs, mainly propionic acid (C3) (*p* = 0.0001) and isovaleric acid (iC5) (*p* = 0.014). FRE dogs also had a lower proportion of C15:0 (*p* = 0.0003), C16:1n-9 (*p* = 0.0095), C16:1n-7 (*p* = 0.0001), C20:5n-3 (*p* = 0.0034) and monounsaturated fatty acids (*p* = 0.0315), and tended to have lower n-3 (*p* = 0.058) and a reduced desaturase activity index in the stool when compared with the control group. However, the dogs with chronic enteropathy tended to have greater C20:4n-6 (*p* = 0.065) in their faeces as signs of damage at the intestinal level. The faecal parameters were better predictors than plasma. The highest correlations between faecal odd-chain, medium- or long-chain fatty acids and SCFAs were observed for C15:0 that correlated positively with faecal acetic acid (C2) (r = 0.72, *p* = 0.004), propionic acid (r = 0.95, *p* = 0.0001), isobutyric acid (iC4) (r = 0.59, *p* = 0.027) and isovaleric acid (r = 0.64, *p* = 0.0136), as well as with total SCFAs (r = 0.61, *p* = 0.02). Conversely, faecal C20:4n-6 showed a high inverse correlation (r = −0.83, *p* = 0.0002) with C2 and C3 (r = −0.59, *p* = 0.027). Canine inflammatory bowel disease (IBD) activity (CIBDAI) index correlated negatively mainly with faecal measurements, such as C3 (r = −0.869, *p* = 0.0005) and C15:0 (r = −0.825, *p* = 0.0018), followed by C16:1/C16:0 (r = −0.66, *p*= 0.0374) and iC5 (r = −0.648, *p* = 0.0310), which would indicate that these fatty acids could be good non-invasive indicators of the chronic inflammatory status, specifically FRE.

## 1. Introduction

Chronic enteropathy (CE) is a very common diagnosis given for dogs with chronic digestive signs, with an estimated prevalence of 70% in cases with chronic diarrhoea [1]. CE can be further subdivided retrospectively by the response to treatment into food-responsive enteropathy (FRE), antibiotic-responsive enteropathy (ARE), immunosuppressant-responsive enteropathy (IRE) and non-responsive enteropathy (NRE) [2]. Several retrospective studies suggested that FRE is probably the most common CE in dogs, with a prevalence greater than 60–70% of cases [1,3]. The significance of the effect of diet in different subtypes of CE is increasing. In fact, it was recently described that dogs with protein-losing enteropathy with a previous non-response to a combination of dietary therapies, glucocorticoids and immunosuppressive medications can achieve remission following a dietary change [4,5,6].

During recent years, the scientific focus has been directed to identifying biomarkers of interest in prognosis and treatment. However, to date, there are no tests that can replace treatment trials [7,8,9,10].

Short-chain fatty acids (SCFAs) are major end products of dietary fibre formed by bacteria [11]. Their role in health and disease was previously evaluated, taking into account their relationship with digestive microbiota and their effects on the immune system and gastrointestinal motility [12,13,14]. Previous studies in human medicine have shown a decrease in faecal concentrations of SCFAs in different chronic digestive diseases, such as inflammatory bowel disease [15]. Similar results were found in human populations with a high risk of colon cancer [16]. In veterinary sciences, the effect of dietary change or intervention on SCFAs was widely evaluated in healthy dogs [17,18,19], but information about faecal SCFAs in different canine digestive diseases is limited. To the best of our knowledge, systematic evaluation of faecal SCFAs has only been performed in dogs with acute diarrhoea [20] and CE [21].

In addition, some studies revealed the potential interest of other fatty acids present in faeces as diagnostic indicators of certain chronic intestinal diseases. This is due to the important functions that lipids have in the body, from being part of cellular structures to being a source of energy, participating in metabolic regulation or as precursors of certain substances [22]. Thus, DePreter et al. [23] found that faecal medium-chain fatty acids were decreased in human patients with inflammatory intestinal diseases and considered hexanoate levels as a good tool for gut disease prediction. Song et al. [24] observed a certain relationship between humans with colorectal cancer and the amount of monounsaturated or polyunsaturated fatty acids in the stool, although there were contradictory results when associating certain long-chain fatty acids with the appearance of disease. Furthermore, some of these fatty acids, such as odd-chain fatty acids (OCFA), were described as coming mainly from gut-derived propionic acid synthesised endogenously in the body [25]. Hence, disorders of propionate were detected through plasma total odd-chain fatty acids determination [26]. OCFA was also used as a potential indicator of rumen function, as well as bacterial matter [27]; however, in non-ruminant species, OCFA are present in small proportions [28]. Recent studies carried out in humans show that plasma and tissue OCFA may be associated not only with lipid status in the organism [28] but also with the gut microbiota [29]. However, there is a lack of information on the diagnostic utility of the fatty acid profile (SCFAs, OCFAs or long-chain fatty acids) in the stool or plasma in chronic intestinal diseases in dogs. In addition, some studies reported that long-chain fatty acids could be involved in the development and treatment of some chronic inflammatory diseases [30]; therefore, the complete fatty acid profile in faeces and plasma as non-invasive procedures deserve more attention. 

Thus, the aim of this study was to evaluate differences in SCFAs and the total fatty acid profile of faeces and plasma as possible indicators of inflammatory chronic disease in the dog, specifically food-responsive enteropathy, and to study the possible correlation between SCFAs as indicators of gut homeostasis and the fatty acid profile in order to determine the most adequate fatty acid for intestinal disease prediction.

## 2. Materials and Methods

### 2.1. Animals and Sample Collection

Healthy dogs included in the study did not have any clinical signs, including digestive signs, within the past 4 months before the sample collection. Physical examinations and routine bloodwork of these dogs had to be normal to be enrolled in the study. Asymptomatic dogs with chronic diseases were excluded from the study.

The criteria for the inclusion of sick dogs in the study included the persistence of clinical signs of chronic digestive disease (vomiting, diarrhoea, weight loss or anorexia/hyporexia) for at least 3 weeks. All dogs included in the study had a favourable response to an elimination diet (hydrolised protein or novel protein diet) after one month. Based on the response to dietary therapy, the disease of these dogs was classified as FRE. No dog included in the study had protein-losing enteropathy. 

Signalment, including age, sex, breed, sexual status, body weight and body condition score, was collected for every dog. Information about specific clinical signs related to chronic digestive disease was obtained in order to calculate the chronic enteropathy activity index (CIBDAI), as previously described [31].

Faecal samples were collected by the owners after spontaneous defecation and received at the clinic in less than 3 h, where they were immediately frozen at −20 °C until analysis. Blood (2 mL) extracted using jugular or cephalic venipuncture were collected in heparine tubes. Plasma obtained after subsequent centrifugation was stored at −80 °C. Faecal and blood samples of dogs with FRE were collected before starting the dietary treatment. The pre-experimental diet for all dogs was mainly based on cereals, animal proteins and vegetable/animal fats (averaged percentages according to the manufacturer’s composition: humidity, 9.5 ± 0.0; crude protein, 26.8 ± 3.4; crude fat, 11.7 ± 4.4; ash, 5.9 ± 1.7; crude fibre, 1.8 ± 0.5; soluble fibre, 6.0 ± 0.7; nitrogen-free extractives, 38.3 ± 12.5; Ca, 0.9 ± 0.1; *p*, 0.7 ± 0.1; C18:2, 2.9 ± 1.1; ∑n-6, 2.7 ± 1.2; ∑n-3, 0.7 ± 0.1; mg/kg vitamin E: 619.2 ± 245.5; metabolic energy/100 g: 3332.3 ± 645.2. No vitamins, minerals, energy or any other supplements were administered at least 4 days before sample collection. 

Their participation in the study was always carried out through the informed consent of the owners. All procedures and protocols were approved by the Animal Research Committee of the Veterinary Medicine Teaching Hospital, Complutense University of Madrid (reference number 11/2021). 

### 2.2. Laboratory Analysis

#### 2.2.1. Concentration of Vitamin E in Plasma Samples

The concentration of vitamin E in the plasma samples was quantified as described elsewhere [32]. Vitamin E (α-tocopherol) was extracted directly without saponification. Duplicate plasma aliquots were mixed with a dibasic sodium phosphate buffer (0.054 M) adjusted to pH 7.0. Tocopherol was extracted via centrifugation (600× *g* for 10 min at 4 °C) after the addition of hexane to the mixture. After the evaporation of the upper layer, the remaining residue was dissolved in ethanol. Tocopherol was analysed using reverse-phase HPLC (HP 1100, equipped with a diode array detector) (Agilent Technologies, Waldbronn, Germany) [32]. Identification was carried out using the pure compound (Sigma-Aldrich, Alcobendas, Madrid) and quantification (µg of α-tocopherol per mL of plasma) was carried out by means of a standard curve built with the pure compound.

#### 2.2.2. Analysis of Short-Chain Fatty Acids in Faecal Samples

Determination of short-chain fatty acids in the faecal samples was carried out as previously described [33]. Frozen dried stool samples were accurately weighed in a 2 mL safe-lock micro test tube. Two glass balls (2 mm Ø) and 1.0 mL distilled water were added. After being tightly capped, the tubes were placed on the adapters and homogenised for 5 min at 30 Hz in a Mixer Mill MM400 (Retsch technology, Haan, Germany). The final system was allowed to separate via centrifugation (10 min, 10,000 rpm). The extraction was repeated three times. Then, the faecal suspension was transferred into a vial and the internal standard (20 mM 4-methylvaleric acid solution) was spiked and the pH was adjusted to 2–3 by adding 25% phosphoric acid. Finally, this solution was placed in vials for gas chromatography injection. Chromatographic analysis was carried out using an Agilent 6850N GC system equipped with a flame ionisation detector (FID) (Agilent Technologies, Waldbronn, Germany). A fused-silica capillary column with a free fatty acid phase (DB-FFAP 125-3237, J&W Scientific, Agilent Technologies Inc., Santa Clara, CA, USA) of 30 m × 0.53 mm i.d. coated with a 0.50 µm thickness film was used. Nitrogen was used as the carrier gas at a constant pressure of 15 psi. The initial oven temperature was 100 °C maintained for 0.5 min, raised to 180 °C at 8 °C/min and held for 1.0 min, then increased to 200 °C at 20 °C/min and finally held at 200 °C for 5 min. The temperatures of the FID and the injection port were 240 °C and 200 °C, respectively. The flow rates of hydrogen, air and nitrogen as makeup gases were 40, 300 and 30 mL/min, respectively. Data handling was carried out with HP ChemStation Plus software (Agilent Technologies, Waldbronn, Germany). Identification and quantification were carried out using pure standards (Sigma-Aldrich, Alcobendas, Spain). An aqueous stock standard solution was prepared for each acid with a concentration of 400 mM for acetic acid, propionic acid and n-butyric acid; 200 mM for n-valeric acid and i-valeric acid; 100 mM for i-butyric acid; 50 mM for n-caproic acid; and 15 mM for n-heptanoic acid.

#### 2.2.3. Extraction of Total Fat and Fatty Acid Profile of Plasma and Faecal Samples

Plasmatic and faecal total lipids were extracted and then analysed for fatty acid profile determination. A solvent mixture of dichloromethane-methanol 8:2 was added to lyophilised weighted samples (Lyoquest, Telstar, Tarrasa, Spain) and after homogenisation in a mixer mill (MM400, Retsch technology, Haan, Germany) and centrifugation (8 min at 10,000 rpm), the upper layer containing lipids were collected. The lipid content was quantified gravimetrically after evaporation of the solvent in a nitrogen stream [34]. Fatty acid methyl esters (FAMEs) were obtained by heating the lipids (80 °C for 1 h) in the presence of methanol:toluene:H_2_SO_4_ (88:10:2 by volume), as described elsewhere [35]. After esterification, FAMEs were extracted with hexane and separated in a gas chromatograph (HP 6890 Series GC System; Hewlett Packard, Avondale, PA, USA) after direct injection of the sample. The gas chromatograph was provided with an automatic injector (hold at 170 °C), a flame ionisation detector (hold at 250 °C) and a capillary column (HP-Innowax polyethylene glycol, 30 m × 0.316 mm × 0.25 µm). After injection, the oven temperature was increased to 210 °C at a rate of 3.5 °C/min, then to 250 °C at a rate of 7 °C/min [35]. Identification and quantification of the FAMEs were made by comparing the retention times with those of authentic standards (Sigma–Aldrich, Alcobendas, Spain). Results were expressed as grams per 100 grams of quantified fatty acids.

Different indices were measured to estimate the desaturase or elongase activities.

The Δ9 desaturase index was calculated as the ratio of C18:0 to C18:1n-9 and as the ratio of C16:0 to C16:1.

The elongase index was calculated as the ratio of C18:0 to C16:0 and as the ratio of C20:5 to C22:5.

### 2.3. Statistical Analysis

Data were analysed following a completely randomised design using the general linear model (GLM) procedure contained in SAS (version 9; SAS Inst. Inc., Cary, NC, USA).

Data were presented as the mean of each group and the standard error of the mean (SEM), together with significance levels (*p*-values). Tukey’s test was used to separate the treatment means. The differences between means were considered statistically significant at *p* < 0.05. Pearson correlations (among SCFAs and plasma or faecal fatty acids, or CIBDAI index and the other variables) were calculated using the Statgraphics-18 program. A linear adjustment between variables was carried out by means of the Statgraphics-18 program (Statgraphics Centurion XVIII, version 18.1.12).

## 3. Results and Discussion

### 3.1. Signalment of Dogs

Data regarding the age, sex, sexual status, body weight, body condition score (BCS) and canine inflammatory bowel disease activity index (CIBDAI) are shown in Table 1. The breeds of dogs with FRE (*n* = 9) were three mongrel dogs and one each of labrador retriever, cocker spaniel, miniature schnauzer, Maltese, short-haired dachshund and chihuahua. The breeds of the healthy dogs (*n* = 6) were 5 mongrel dogs and one Gordon setter. Differences concerning age, body weight or body condition score between the FRE and control groups were not statistically affected. The breed or reproductive status was not evaluated because of the insufficient numbers of individuals to study these effects. Previous studies indicated a higher prevalence of chronic enteropathy in purebreds, such as German shepherd, rottweiler, Weimaraner, border collie, or boxer when compared with mixed-breed dogs [36]. However, based on the study of these factors on the microbiome in dogs with IBD, other authors did not observe changes due to breed or reproductive status, although they did observe changes due to the disease [37].

### 3.2. Oxidative Status and Lipid Plasma Profile of the Dogs

The oxidative status, total fat and fatty acid profile of the plasma are presented in Table 2. The FRE dogs had a lower concentration (*p* = 0.026) of α-tocopherol as an indicator of the oxidative status of the animal, whereas no changes were observed in the proportion of plasma fat. Other authors reported reduced plasma antioxidant concentrations (mainly vitamin E and A) in human patients with inflammatory chronic disease [38]. The pathogenesis studies of inflammatory bowel disease (IBD) revealed that human patients with IBD had an excessive amount of oxidised molecules compared with healthy controls [39,40]. According to Rezaie et al. [39], in order to counteract this state, the organism responds with higher antioxidant production, which reduces the deposits and the capacity of the organism’s response to oxidative stress. Yuksel et al. [41] also reported that total antioxidant status in humans could be a good predictor of IBD. Hence, the use of substances based on the assessment of the redox status was recently proposed as a therapeutic alternative [42,43]. In addition, in rats, a relationship was found between different regions in the gastrointestinal tract and the oxidative status according to the microbiota since some microorganisms are able to produce endogenous antioxidants [40]. It is interesting to highlight that in the present study, the oxidative status, measured as the plasma vitamin E concentration in the alpha-tocopherol form, was also an indicator of greater C3 (r = 0.56, *p* = 0.044) in faeces, and a positive trend was observed for IC4 (r = 0.51, *p* = 0.072) and total SCFAs (r = 0.53, *p* = 0.064). Therefore, the higher the concentration of vitamin E in the plasma, the higher the content of C3 in faeces. There is no previous information on this result in dogs with chronic diseases, but some studies carried out in mice point to a direct connection between SCFAs and the inhibition of oxidative stress and inflammation [44].

Concerning the lipid plasma profile, previous investigations did not find any changes in plasma triglyceride content in human patients with IBD [45]. In the present study, the specific fatty acid profile of the plasma (Table 2) revealed that the FRE dogs had higher C20:3n-6 (*p* = 0.0056) and tended to have higher C18:1n-9 (*p* = 0.072) and C20:2n-6 (*p* = 0.062), although the differences were not statistically significant; meanwhile, these FRE dogs had lower C20:0 (*p* = 0.003), C20:5n-3 (*p* = 0.033), C22:5n-3 (*p* = 0.005), polyunsaturated fatty acids (PUFA) (*p* = 0.021) and n-6 (*p* = 0.041), and tended to have lower proportions of C18:2n-6 (*p* = 0.051) and n-3 (*p* = 0.056), although these last two were not statistically different when compared with the control group. Other authors [46,47,48] reported decreased polyunsaturated fatty acids in different intestinal chronic diseases. In a detailed study carried out in humans, Esteve-Comas et al. [48] found a relationship between the severity of the disease (ulcerative colitis and Crohn’s disease) and the degree of decrease in n-3 and n-6 fatty acids. These authors also observed an increase in monounsaturated fatty acids in both diseases corresponding to the status severity. Similarly, Kuroki et al. [47] found negative correlations between the Crohn’s disease activity index and serum polyunsaturated fatty acids. The main essential polyunsaturated fatty acids come mainly from the diet, while monounsaturated fatty acids can be synthesised endogenously in the body. A lower proportion of polyunsaturated fatty acids could be related to malabsorption processes, or to a higher lipolytic activity of these fatty acids that are used preferentially for energy supply [49] as structural parts of cell membranes or as precursors of inflammation-regulating substances [50] in a high-requirement status, such as chronic digestive disease [47], consequently resulting in a higher proportion of other plasma fatty acids. In addition, a predominance of monounsaturated fatty acids due to desaturation phenomena to obtain energy in metabolic states in which there was a significant decrease in polyunsaturated fatty acids was described [22,49]. Conversely, in humans with ulcerative colitis, Bazarganipour et al. [51] reported increases of EPA and DHA (20:5n-3 and C22:6n-3) in the blood of patients with a more severe stage of the disease since these fatty acids are precursors of resolvins and maresins that are synthesised in order to repair the barrier disruption and were attributed to anti-inflammatory properties; however, dietary supply of these essential fatty acids should have been considered. As indicated by Hengstermann et al. [52], discrepancies in results between studies could be attributed to the malnutrition status and dietary therapeutic alternatives used to counteract the disease state. Furthermore, in the present research, it is interesting to highlight the greater proportions of some n-6 fatty acids (C20:3n-6 and C20:2n-6) observed in plasma from FRE dogs when compared with the control dogs, contrary to the decrease in n-6 fatty acids observed by other authors [47]. It was described that C20:3n-6 is the immediate precursor of PGE1 and C20:4n-6 (the main component of the phospholipids membranes) [22], and C20:3n-6 can be obtained directly from C20:2n-6 via Δ8-desaturase in a direct alternative route that is mainly activated with high eicosanoid requirements, such as in inflammation [53]. This is the first study in which the plasma fatty acid profile of dogs with inflammatory digestive chronic diseases was studied, specifically in dogs with food-responsive CE. The specific increase in these long-chain n-6 fatty acids (C20:2 and C20:3) in the blood could indicate their diagnostic potential as indicators of inflammation.

### 3.3. Short-Chain Fatty Acid (SCFA) Profile in Faecal Samples

In the present research, the short-chain fatty acid profile of faecal samples was also quantified (Table 3). Dogs with FRE showed a decrease in faecal levels of SCFAs. Similar results were found in dogs with different acute and chronic digestive diseases [20,21]. It was suggested that decreased SCFAs might contribute to the inflammatory status of these disorders [21]. Intestinal microbiota of dogs with CE showed a decrease in the phylum Firmicutes, especially in Clostridium XIVa and IV, which are significant producers of SCFAs [54,55].

When analysing the specific content of SCFAs in the present study, the propionic acid concentration decreased in the dogs with FRE. A similar decrease in the concentrations of faecal propionic acid was also found in dogs with acute diarrhoea [20] and in dogs with CE [21]. These similar results were not unexpected, taking into account the fact that the previous study performed in dogs with CE [21] included some dogs with FRE, but also dogs with other types of CE. The significance of the difference in faecal propionate concentrations found in our study when comparing healthy dogs and dogs with FRE was the most prominent among all SCFAs, similarly to what was previously found in dogs with CE [21]. Propionate can play a role in the pathogenesis of chronic intestinal inflammation in dogs [21]. Its role in the immune system and intestinal inflammation has been widely evaluated, especially in vivo and in rodents. Among these functions, propionate is able to regulate the size and function of the colonic Treg cells that express the transcription factor Foxp3 and protect against colitis in mice [56]. The decreased levels of propionate in dogs with different CE could be potentially significant, taking into account the fact that dogs with IBD have decreased numbers of Foxp3-positive Treg cells in the duodenal mucosa [57,58].

Concerning other SCFAs, information in the literature about faecal branched-chain fatty acids (BCFAs) in dogs is very limited. The relative concentration of isovalerate was associated with increased colitis and the IL-1β concentration of the intestinal mucosa in experimental models of IBD [59]. Our study showed that dogs with FRE also had lower concentrations of faecal isovaleric acid (*p* = 0.014), similar to what was found in rodent models of colitis [60]. Conversely, Guard et al. [20] reported that dogs with acute diarrhoea have similar levels of BCFA to healthy dogs. In contrast, in the present research, faecal butyric acid in dogs with FRE was not altered in comparison with healthy dogs. Similar results were found in dogs with chronic intestinal disease [21], while dogs with acute diarrhoea had higher levels of faecal butyric acid [20]. It was hypothesised that these results could be due to a reduction in the utilisation of butyrate by epithelial cells or a loss into the intestinal lumen in dogs with chronic intestinal inflammation [21].

### 3.4. Total Fatty Acid Profile in Faecal Samples

The total fatty acid profile of faeces from dogs affected with intestinal chronic disease, specifically FRE, or the control group is presented in Table 4. The FRE dogs had a lower proportion of C15:0 (*p* = 0.0003), C16:1n-9 (*p* = 0.0095), C16:1n-7 (*p* = 0.0001), C20:5n-3 (*p* = 0.0034) and monounsaturated fatty acids (MUFA) (*p* = 0.0315), and tended to have lower ∑n-3 (*p* = 0.058) when compared with the control group. However, the FRE dogs had a greater proportion of C18:0 (*p* = 0.017) and tended to have greater C20:4n-6 (*p* = 0.065) in faeces. This fatty acid profile was different from that found in plasma samples; however, some results were connected with those observed in blood, such as the proportion of ∑n-3 fatty acids. It is also interesting to highlight the high proportion of arachidonic acid (C20:4n-6), which could be the result of excessive membrane destruction in sick dogs or to greater production of this fatty acid to repair cellular damage at the intestinal level. This fact coincided with the higher proportions of C20:3n-6 and C20:2n-6 in blood as a faster alternative route for the synthesis of C20:4 [53], which could point to these fatty acids as possible indicators of inflammatory processes.

The lower presence of some fatty acids, such as C15:0, in faecal samples could be in part associated with the different microbiome activity in the digestive system in these sick dogs. It was observed that odd-chain fatty acids (OCFA), such as C15:0 and C17:0, can be endogenously synthesised in the body from short-chain fatty acids, such as propionic acid (C3:0), which mainly come from processes of microbial fermentation in the gut [25]. Sick dogs also had lower levels of MUFA, such as C16:1n-9 and C16:1n-7, in the faeces, which could be explained by the higher metabolic use of these fatty acids, although no significant differences were observed in the blood levels. Other authors reported the high predisposition of C16:1n-7 to undergo β-oxidation [47,61], as well as the preferential use of monounsaturated fatty acids to obtain energy after polyunsaturated fatty acids [35,47]. The poorer utilisation of nutrients and malnutrition associated with animals with inflammatory digestive disease could induce this greater metabolic utilisation rate of certain fatty acids in the present study. Furthermore, the lower presence of these monounsaturated fatty acids and high level of C18:0 in dogs with inflammatory disease could also be explained by a possible lower desaturase activity at the level of the enterocyte membrane. This was confirmed by the lower C16:1/C16:0 and C18:1/C18:0 indices as indicators of the desaturase activity, which were observed in the faecal samples from the sick dogs. Garg et al. [62] reported that although the activity of desaturases in the intestine was lower than in the liver, it had an important effect on the properties of the enterocyte membrane. Therefore, according to the results of the present study, a greater alteration of the intestinal cell membrane could affect such a desaturation capacity. However, it is very interesting to observe in the present study how the elongase capacity found in the intestinal sample was higher in sick dogs. This was more marked in the case of the elongase activity of monounsaturated fatty acids C16:0/C18:0 than in the elongase activity of polyunsaturated fatty acids C22:5/C20:5. Oreshko et al. [63] reported a tendency towards increased serum elongase activity in human patients with celiac disease when compared with healthy controls. The diseased animals probably adapted their metabolic pattern to achieve a greater synthesis of saturated and long-chain polyunsaturated fatty acids as precursors of other energy-supplier fatty acids and main constituents of the membrane structure [63], as well as the synthesis of compounds with anti-inflammatory characteristics.

### 3.5. Correlations between Fatty Acids and SCFAs

There is no previous information on the global diagnostic possibilities of SCFAs, plasma or faeces fatty acid profile for FRE in dogs; therefore, this is the first study in which these compounds were evaluated in healthy dogs and dogs with food-responsive chronic enteropathy. Since some of these measurements are considered of interest in humans, we looked for possible relationships between the different compounds. In the faecal SCFAs, the greater correlations were detected for propionic acid (C3), isobutyric acid (IC4) and isovaleric acid (IC5), followed by acetic acid (C2) and valeric acid (C5) (Table 5). The faecal fatty acids that presented the greatest correlations were C15:0, C16:1n-7 and C20:5n-3, followed by C18:0, MUFA and C20:4n-6. The highest correlations between faecal OCFA, medium- or long-chain fatty acids and SCFAs were observed for C15:0, which correlated positively with C2 (r = 0.72, *p* = 0.004), C3 (r = 0.95, *p* = 0.0001), IC4 (r = 0.59, *p* = 0.027) and IC5 (r = 0.64, *p* = 0.0136), as well as with total SCFAs (r = 0.61, *p* = 0.02). Conversely, C20:4n-6 showed a high inverse correlations with C2 (r = −0.83, *p* = 0.0002) and C3 (r = −0.59, *p* = 0.027). C16:1n-7 correlated positively with C3 (r = 0.85, *p* = 0.0001), IC4 (r = 0.66, *p* = 0.0102) and IC5 (r = 0.72, *p* = 0.0037); meanwhile, C20:5 correlated to a lesser extent with C3 (r = 0.62, *p* = 0.017), IC4 (r = 0.55; *p*= 0.042) and IC5 (r = 0.68, *p* = 0.0072). These fatty acids could therefore be considered as indicators of the intestinal health status and mucosa integrity.

On the other hand, faeces moisture and fat were negatively correlated with SCFA (Table 5). A higher number of correlations were observed for faeces moisture than for fat. Hence, faeces moisture correlated negatively with C3 (r = −0.68, *p* = 0.0077), IC4 (r = −0.55, *p* = 0.040) and IC5 (r = −0.56, *p* = 0.035), and showed a greater correlation for total SCFAs (r = −0.80, *p* = 0.0007) than the other variables. Other authors found a greater SCFA proportion and faecal moisture in the colons of healthy mice [64]; however, in the present research sick dogs were affected with diarrhoea and higher moisture in the stool could be associated with a clinical sign of disease. Therefore, according to the present results and as stated before, C3 could be a good indicator of chronic diarrhoea, but more research is needed in order to know if this compound could be affected between different CE.

The correlations between SCFA and plasma fatty acids, plasma fat and tocopherol concentration were also determined (Table 6). A lower number of significant correlations were observed in the plasma than in the faeces. IC4 and IC5, followed by C3, were the SCFAs that had the greatest number of correlations with the other plasma variables. IC4 correlated negatively with C18:0 (r = −0.61, *p* = 0.025) and positively with C18:3n-3 (r = 0.64, *p* = 0.018), C20:1n-9 (r = 0.57, *p* = 0.042), C20:5n-3 (r = 0.60, *p* = 0.031), C22:4n-6 (r = 0.68, *p* = 0.010) and total n-3 (r = 0.61, *p* = 0.025). In the same way, IC5 correlated positively with C20:0 (r = 0.56, *p* = 0.046), C20:5n-3 (r = 0.68, *p* = 0.010), C22:5n-6 (r = 0.68, *p* = 0.011), total PUFA (r = 0.56, *p* = 0.048) and n-3 (r = 0.59, *p* = 0.013). According to these results, plasma n-3 fatty acids levels could be interesting predictors of intestinal health, probably in connection with the nutritional status of the animal.

Finally, the correlation between BCS and CIBDAI scores and the other parameters of plasma and faeces were also evaluated (Table 7). The significant correlations were mainly detected in faecal parameters. Moreover, the CIBDAI score presented a higher number of significant correlations than BCS. Hence, the CIBDAI score correlated negatively mainly with faecal measurements, such as C3 (r = −0.869, *p* = 0.0005) and C15:0 (r = −0.825, *p* = 0.0018), followed by C16:1/C16:0 (r = −0.66, *p* = 0.0374) and iC5 (r = −0.648, *p* = 0.0310), as well as the sum of C2 + C3 (r = −0.659, *p*= 0.0273) and the sum of C2 + C3 + C4 (r = −0.632, *p* = 0.0369). In addition, the CIBDAI score was also positively correlated with faeces moisture (r = 0.790, *p*= 0.0039). This index is considered a reliable measure of inflammatory activity in canine IBD [31]; therefore, according to the results of the present study, the CIBDAI score would be a good indicator of chronic inflammatory status. Its quantification, together with C3, other or total SCFAs, C15:0 or desaturase capacity in faecal samples would reinforce this non-invasive diagnosis technique in dogs with chronic inflammatory diseases.

## 4. Conclusions

In conclusion, the dogs with FRE had a lower oxidative status and higher plasma proportions of C20:2 and C20:3 as indicators of chronic inflammation, as well as lower propionic acid and branched-chain fatty acids, such as isovaleric acid, in their stools. The short-chain fatty acids correlated better with the total fatty acid profile of the faeces. The high correlations observed between most of the SCFAs and OCFA, such as C15:0, in the faeces indicates the diagnostic potential of this compound. Sick dogs also showed signs of damage at the intestinal level with a greater presence of arachidonic acid (C20:4), as well as a reduced desaturase activity in the stool. Further studies would be warranted in order to elucidate whether specific profiles of faecal SCFAs, OCFA or long-chain fatty acids could be found in dogs with different CEs, such as FRE, ARE and IRE.

## Figures and Tables

**Table 1:** Data of signalment and CIBDA index (chronic enteropathy activity index) in the dogs with FRE (food-responsive enteropathy) and control dogs included in the study.

	FRE Average ± SD (Range)		Control Average ± SD (Range)		*p* ^1^
Age (years)	6.2 ± 3.6	(3–13)	4.5 ± 2.1	(3–6)	NS ^2^
Male	4 (2 entire, 2 castrated)		3 (3 entire)		
Female	5 (1 entire, 4 spayed)		3 (3 spayed)		
Body weight	11.4 ± 9.4	(2.3–31)	15.5 ± 0.7	(15–16)	NS
Body condition score (scale 9)	4.2 ± 1.4	(2–6)	4.5 ± 0.40	(4–5)	NS
CIBDAI index	7 ± 1.8	(5–10)	0	(0–0)	0.0001

^1^*p*: differences were statistically significant when *p* < 0.05; ^2^ NS: not significant.

**Table 2:** Concentration of α-tocopherol, fat and fatty acid profile of plasma samples from the FRE (food-responsive enteropathy) and control dogs.

	FRE		Control		SEM ^6^	*p* ^7^
Plasma α-tocopherol (µg/g)	24.61	^b^	44.62	^a^	3.431	0.0266
Plasma total fat (%)	24.59		29.99		1.630	0.1746
Plasma fatty acids (g per 100 g)						
C14:0	0.35		0.34		0.041	0.9139
C14:1	0.05		0.03		0.010	0.3887
C15:0	0.14		0.16		0.016	0.5581
C16:0	15.00		15.78		0.399	0.4115
C16:1n-9	0.57		0.41		0.060	0.2655
C16:1n-7	1.36		1.13		0.119	0.4007
C17:0	0.40		0.48		0.034	0.3097
C17:1	0.19		0.21		0.011	0.3325
C18:0	18.22		17.67		0.569	0.6785
C18:1n-9	16.77		11.29		1.208	0.0721
C18:1n-7	2.51		2.50		0.078	0.9412
C18:2n-6	22.81		27.72		0.983	0.0511
C18:3n-6	0.27		0.23		0.022	0.4109
C18:3n-3	0.49		0.43		0.083	0.7858
C18:4n-3	0.17		0.15		0.024	0.6721
C20:0	0.09	^b^	0.14	^a^	0.006	0.0032
C20:1n-9	0.18		0.15		0.018	0.5024
C20:2	0.37		0.26		0.023	0.0627
C20:3n-6	0.94	^a^	0.49	^b^	0.058	0.0056
C20:4n-6	14.59		14.33		0.794	0.8886
C20:5n-3	1.39	^b^	2.80	^a^	0.254	0.0335
C22:4n-6	1.84		1.78		0.247	0.9142
C22:5n-3	0.42	^b^	0.73	^a^	0.039	0.0053
C22:6n-3	0.88		0.81		0.053	0.5579
∑SAT ^1^	34.20		34.57		0.689	0.8196
∑MUFA ^2^	21.63		15.71		1.336	0.0783
∑PUFA ^3^	44.17	^b^	49.72	^a^	0.910	0.0217
∑n-6 ^4^	40.45	^b^	44.54	^a^	0.775	0.0410
∑n-3 ^5^	3.35		4.92		0.321	0.0560
∑n-6/∑n-3	13.63		9.12		1.180	0.1218

^1^ ∑SAT: sum of total saturated fatty acids; ^2^ ∑MUFA: sum of total monounsaturated fatty acids; ^3^ ∑PUFA: sum of total polyunsaturated fatty acids; ^4^ ∑n-6: sum of total n-6 fatty acids; ^5^ ∑n-3: sum of total n-3 fatty acids; ^6^ SEM: standard error of the mean; ^7^ *p*: differences were statistically significant when *p* < 0.05. Values with different superscripts (a,b) were statistically significant.

**Table 3:** Faecal parameters and concentration of short-chain fatty acids (SCFAs) in the faeces from the FRE (food-responsive enteropathy) and control dogs.

	FRE		Control		SEM ^1^	*p* ^2^
*Faecal parameters*						
Moisture (%)	68.912	^a^	62.138	^b^	1.271	0.0437
Fat (%)	3.619		2.403		0.288	0.0975
Fat (% DM)	11.965		6.332		1.118	0.0541
*Short-Chain Fatty Acids* (mmol/g DM)						
Acetic acid	1.901	^b^	3.852	^a^	0.312	0.0219
Propionic acid	0.400	^b^	3.049	^a^	0.128	0.0001
Isobutyric acid	0.236	^b^	0.939	^a^	0.128	0.0386
Butyric acid	1.454		1.634		0.419	0.8562
Isovaleric acid	0.074	^b^	0.387	^a^	0.042	0.0089
Valeric acid	0.723		0.000		0.469	0.5196
Total SCFAs	4.787	^b^	9.862	^a^	0.922	0.0384
∑C2,C3 ^3^	2.300	^b^	6.901	^a^	0.379	0.0004
∑C2,C3,C4 ^3^	3.754	^b^	8.535	^a^	0.560	0.0041
∑IC4,IC5 ^3^	0.310	^b^	1.326	^a^	0.141	0.0108

^1^ SEM: standard error of the mean; ^2^ *p*: differences were statistically significant when *p* < 0.05. Values with different superscripts (a,b) were statistically significant; ^3^ C2: acetic acid; C3: propionic acid; C4: butyric acid; IC4: isobutyric acid; IC5: isovaleric acid.

**Table 4:** Fatty acid profile (g/100 g of total fatty acids) in the faeces from the FRE (food-responsive enteropathy) and control dogs.

	FRE		Control		SEM ^6^	*p* ^7^
C14:0	1.499		2.131		0.244	0.2212
C14:1	0.052		0.055		0.010	0.6906
C15:0	0.341	^b^	1.169	^a^	0.064	0.0003
C16:0	24.685		25.545		1.055	0.5925
C16:1n-9	0.253	^b^	0.313	^a^	0.008	0.0095
C16:1n-7	1.526	^b^	2.955	^a^	0.094	0.0001
C17:0	0.653		0.564		0.105	0.8873
C17:1	1.129		1.058		0.111	0.8607
C18:0	17.264	^a^	11.677	^b^	0.744	0.0172
C18:1n-9	20.931		22.557		1.050	0.7389
C18:1n-7	5.506		5.385		0.716	0.8425
C18:2n-6	14.784		16.953		1.794	0.9021
C18:3n-6	0.096		0.083		0.010	0.6815
C18:3n-3	1.178		1.383		0.143	0.7735
C18:4n-3	0.373		0.480		0.041	0.1855
C20:0	0.491		0.420		0.065	0.9366
C20:1n-9	0.659		0.444		0.051	0.2151
C20:2	0.353		0.465		0.029	0.1096
C20:3n-6	1.010		1.017		0.148	0.7916
C20:4n-6	3.707		1.436		0.421	0.0657
C20:5n-3	0.448	^b^	0.816	^a^	0.043	0.0034
C22:4n-6	0.482		0.353		0.067	0.5617
C22:5n-3	0.510		0.490		0.058	0.9505
C22:6n-3	2.068		2.253		0.164	0.354
∑SAT ^1^	44.934		41.506		2.073	0.6699
∑MUFA ^2^	30.058	^b^	32.767	^a^	0.428	0.0315
∑PUFA ^3^	25.009		25.727		1.925	0.9294
∑n-6 ^4^	20.079		19.840		1.913	0.7381
∑n-3 ^5^	4.577		5.422		0.200	0.0584
C18:1/C18:0	1.212		1.932		0.112	0.0584
C16:1/C16:0	0.062	^b^	0.116	^a^	0.004	0.0005
C20:4n-6/C20:2n-6	11.219		3.130		1.554	0.0536
Elongase C16/C18	0.698	^a^	0.458	^b^	0.024	0.0023
Elongase C225/C205	1.206		0.612		0.123	0.0693

^1^ ∑SAT: sum of total saturated fatty acids; ^2^ ∑MUFA: sum of total monounsaturated fatty acids; ^3^ ∑PUFA: sum of total polyunsaturated fatty acids; ^4^ ∑n-6: sum of total n-6 fatty acids; ^5^ ∑n-3: sum of total n-3 fatty acids; ^6^ SEM: standard error of the mean; ^7^ *p*: differences were statistically significant when *p* < 0.05. Values with different superscripts (a,b) were statistically significant.

**Table 5:** Correlation coefficients between short-chain fatty acids (SCFAs) and the fatty acid profile in faeces.

Faeces	Acetic Acid		Propionic Acid		Isobutyric Acid		Butyric Acid		Isovaleric Acid		Valeric Acid		Total SCFAs	
Fat	−0.39		−0.61		−0.47		−0.24		−0.23		0.02		−0.55	
Moisture	−0.21		−0.68	^b^	−0.55	^a^	−0.50		−0.56	^a^	−0.38		−0.80	^b^
C14:0	0.46		0.10		−0.09		−0.41		−0.11		−0.35		−0.10	
C14:1	0.26		−0.24		−0.35		−0.24		−0.36		−0.15		−0.21	
C15:0	0.72	^b^	0.95	^b^	0.59	^a^	−0.05		0.64	^a^	−0.34		0.61	^a^
C16:0	0.49		0.34		0.08		0.04		0.08		−0.32		0.22	
C16:1n-7	0.46		0.85	^b^	0.66	^a^	0.07		0.72	^b^	−0.29		0.55	^a^
C16:1n-9	0.36		0.25		0.10		−0.22		0.12		−0.05		0.16	
C17:1	−0.43		0.21		0.41		0.38		0.30		0.33		0.26	
C18:0	−0.33		−0.70	^b^	−0.32		−0.21		−0.73	^b^	−0.08		−0.60	^a^
C18:1n-7	0.19		0.02		0.19		0.01		−0.13		−0.11		0.06	
C18:1n-9	−0.03		0.10		−0.14		−0.15		0.23		−0.12		−0.09	
C18:2n-6	−0.10		0.02		−0.04		0.23		0.21		0.38		0.21	
C18:3n-3	0.02		0.00		−0.25		−0.32		−0.02		−0.12		−0.20	
C18:3n-6	−0.55	^a^	−0.11		−0.20		0.42		0.04		0.31		−0.01	
C18:4n-3	−0.05		0.50		0.35		0.36		0.16		−0.03		0.35	
C20:0	−0.23		0.07		0.21		−0.16		−0.10		−0.24		−0.20	
C20:1n-9	−0.30		−0.42		−0.30		−0.15		−0.54		−0.07		−0.44	
C20:2n-6	0.42		0.55	^a^	0.35		−0.31		0.25		−0.50		0.12	
C20:3n-6	−0.08		0.16		−0.22		0.49		0.05		0.60	^a^	0.43	
C20:4n-6	−0.83	^b^	−0.59	^a^	0.00		0.08		−0.28		0.36		−0.39	
C20:5n-3	0.21		0.62	^a^	0.55	^a^	−0.18		0.68	^b^	−0.28		0.26	
C22:4n-6	0.09		−0.03		0.00		0.34		−0.03		0.25		0.25	
C22:5n-3	−0.12		−0.17		0.02		−0.05		0.14		0.28		−0.01	
C22:6n-3	−0.10		0.37		0.58	^a^	−0.07		0.40		−0.24		0.09	
∑SAT ^1^	0.23		−0.11		−0.11		−0.18		−0.34		−0.32		−0.19	
∑MUFA ^2^	0.26		0.59	^a^	0.34		−0.18		0.56	^a ^	−0.42		0.17	
∑PUFA ^3^	−0.33		−0.06		0.01		0.26		0.20		0.48		0.15	
∑n-6 ^4^	−0.09		−0.33		−0.13		−0.05		0.29		0.12		0.51	
∑n-3 ^5^	−0.05		0.44		0.45		−0.22		0.51		−0.21		0.08	

^1^ ∑SAT: sum of total saturated fatty acids; ^2^ ∑MUFA: sum of total monounsaturated fatty acids; ^3^ ∑PUFA: sum of total polyunsaturated fatty acids; ^4^ ∑n-6: sum of total n-6 fatty acids; ^5^ ∑n-3: sum of total n-3 fatty acids. ^a^ Significant at <0.05 probability level (blue color); ^b^ significant at <0.01 probability level (red color).

**Table 6:** Correlation coefficients between short-chain fatty acids (SCFAs) and fatty acid profile in the plasma.

	Acetic Acid		Propionic Acid		Isobutyric Acid		Butyric Acid		Isovaleric Acid		Valeric Acid		Total SCFA
Plasma fat	0.39		0.45		0.39		−0.26		0.68	^a^	−0.11		0.26
Plasma α-toc	−0.08		0.56	^a^	0.36		0.51		0.47		0.21		0.53
C14:0	0.23		0.07		−0.16		0.60	^a^	0.08		0.38		0.48
C15:0	−0.07		0.25		−0.04		−0.09		0.48		−0.17		−0.02
C16:0	0.43		0.09		0.06		−0.31		0.25		−0.38		−0.05
C16:1n-7	−0.03		−0.10		−0.08		0.67	^a^	−0.24		0.52		0.40
C16:1n-9	−0.41		−0.29		0.33		−0.12		−0.39		−0.11		−0.32
C17:0	0.71	^b^	0.39		0.05		0.04		0.24		−0.13		0.39
C17:1	0.25		0.30		0.36		0.39		0.23		0.07		0.41
C18:0	−0.01		−0.07		−0.61	^a^	−0.09		0.13		0.19		−0.07
C18:1n-7	−0.49		0.10		0.27		0.25		0.02		0.20		0.06
C18:1n-9	−0.38		−0.40		−0.10		−0.08		−0.57	^a^	−0.05		−0.38
C18:2n-6	0.38		0.41		0.35		−0.03		0.42		−0.18		0.28
C18:3n-3	−0.19		−0.22		0.64	^a^	−0.15		−0.15		−0.23		−0.21
C20:0	0.29		0.74	^b^	0.36		−0.02		0.56	^a^	−0.28		0.33
C20:1n-9	−0.38		−0.28		0.57	^a^	−0.19		−0.20		−0.28		−0.35
C20:3n-6	−0.54		−0.66	^a^	−0.34		−0.08		−0.53		0.22		−0.46
C20:4n-6	0.12		0.04		−0.45		0.29		0.02		0.44		0.29
C20:5n-3	0.03		0.54		0.60	^a^	0.00		0.68	^a^	−0.29		0.21
C22:4n-6	−0.39		−0.17		0.68	^a^	−0.08		−0.12		−0.06		−0.17
C22:5n-3	0.26		0.62	^a^	0.21		−0.01		0.68	^a^	−0.11		0.33
C22:6n-3	−0.28		−0.35		−0.29		0.30		−0.18		0.37		−0.01
∑SAT ^1^	0.30		0.04		−0.48		−0.22		0.30		−0.06		−0.04
∑MUFA ^2^	−0.39		−0.38		−0.06		−0.01		−0.55		0.00		−0.33
∑PUFA ^3^	0.34		0.48		0.37		0.15		0.56	^a^	0.04		0.46
∑n-6 ^4^	0.43		0.43		0.21		0.19		0.45		0.15		0.51
∑n-3 ^5^	0.00		0.43		0.61	^a^	−0.01		0.59	^a^	−0.26		0.17

^1^ ∑SAT: sum of total saturated fatty acids; ^2^ ∑MUFA: sum of total monounsaturated fatty acids; ^3^ ∑PUFA: sum of total polyunsaturated fatty acids; ^4^ ∑n-6: sum of total n-6 fatty acids; ^5^ ∑n-3: sum of total n-3 fatty acids. ^a^ Significant at <0.05 probability level (blue color); ^b^ significant at <0.01 probability level (red color).

**Table 7:** Correlation coefficients between BCS (body condition score) and CIBDAI (chronic enteropathy activity index) and the other parameters of the faeces from the FRE (food-responsive enteropathy) and control dogs.

	BCS		CIBDAI Index	
	r	*p*-Value	r	*p*-Value
Faeces fat	−0.15	0.6689	0.50	0.1177
Faeces moisture	−0.55	0.0794	0.79	0.0039
Acetic acid (C2)	0.13	0.7091	−0.35	0.2962
Propionic acid (C3)	0.35	0.287	−0.87	0.0005
Isobutyric acid (iC4)	−0.27	0.4192	−0.45	0.1610
Butiryc acid (C4)	0.55	0.0806	−0.13	0.7141
Isovaleric acid (iC5)	0.16	0.637	−0.65	0.0310
Valeric acid (C5)	0.45	0.1688	−0.05	0.8733
∑SCFAs ^1^	0.54	0.0831	−0.59	0.0552
C2 + C3	0.26	0.4413	−0.66	0.0273
C2 + C3 + C4	0.52	0.1011	−0.63	0.0369
IC4 + IC5	−0.18	0.6062	−0.57	0.0668
C14:0	0.23	0.4919	−0.10	0.7644
C14:1	−0.61	0.0451	0.42	0.2040
C15:0	0.35	0.2851	−0.82	0.0018
C16:0	0.43	0.1874	−0.05	0.8833
C16:1n-7	−0.16	0.631	−0.54	0.0865
C16:1n-9	0.18	0.5939	−0.39	0.2402
C17:0	0.53	0.0927	0.02	0.9454
C17:1	0.45	0.1636	−0.46	0.1569
C18:0	0.20	0.549	0.48	0.1306
C18:1n-7	0.29	0.3835	−0.05	0.8912
C18:1n-9	−0.48	0.1371	0.08	0.8243
C18:2n-6	−0.44	0.1737	0.00	0.9924
C18:3n-3	−0.06	0.8692	0.21	0.5412
C18:3n-6	−0.32	0.3362	−0.02	0.9473
C18:4n-3	0.21	0.5391	−0.26	0.4327
C20:0	0.38	0.2493	−0.26	0.4428
C20:1n-9	0.39	0.2302	0.16	0.6400
C20:2	0.20	0.5541	−0.52	0.0997
C20:3n-6	0.64	0.0335	−0.34	0.3052
C20:4n-6	−0.03	0.9385	0.14	0.6802
C20:5n-3	−0.13	0.6926	−0.53	0.0923
C22:4n-6	0.52	0.1031	−0.04	0.8956
C22:5n-3	0.06	0.8668	−0.07	0.8469
C22:6n-3	0.35	0.2869	−0.46	0.1571
∑SAT ^2^	0.43	0.1913	0.13	0.7040
∑MUFA ^3^	−0.44	0.1779	−0.24	0.4781
∑PUFA ^4^	−0.34	0.3049	−0.07	0.8277
∑n-6 ^5^	−0.35	0.2939	0.00	0.9894
∑n-3 ^6^	0.05	0.8747	−0.52	0.0994
C16:1/C16:0	−0.33	0.3536	−0.66	0.0374

^1^ ∑SCFAs: sum of total short-chain fatty acids; ^2^ ∑SAT: sum of total saturated fatty acids; ^3^ ∑MUFA: sum of total monounsaturated fatty acids; ^4^ ∑PUFA: sum of total polyunsaturated fatty acids; ^5^ ∑n-6: sum of total n-6 fatty acids; ^6^ ∑n-3: sum of total n-3 fatty acids.

## Data Availability

Not applicable.
